# E-mental health in Germany — what is the current use and what are experiences of different types of health care providers for patients with mental illnesses?

**DOI:** 10.1186/s13690-023-01150-y

**Published:** 2023-07-17

**Authors:** Elena Caroline Weitzel, Maria Schwenke, Georg Schomerus, Peter Schönknecht, Markus Bleckwenn, Anja Mehnert-Theuerkauf, Steffi G. Riedel-Heller, Margrit Löbner

**Affiliations:** 1 Institute of Social Medicine, Occupational Health and Public Health (ISAP), Medical Faculty, University of Leipzig, Philipp-Rosenthal-Straße 55, Leipzig, 04103 Germany; 2 Department of Psychiatry and Psychotherapy, University of Leipzig Medical Center, Leipzig, Germany; 3 Department of Psychiatry and Psychotherapy, University Affiliated Hospital Arnsdorf, Technical University of Dresden, Dresden, Germany; 4 Department of General Practice, Medical Faculty, University of Leipzig, Leipzig, Germany; 5 Department of Medical Psychology and Medical Sociology, Medical Faculty, University of Leipzig, Leipzig, Germany

**Keywords:** E-mental-health, Health care apps, Implementation, Routine care

## Abstract

**Background:**

As a new and effective support option, e-mental health interventions can be useful in complementing treatment in mental health care. To date, little is known about how health care providers use these programs to treat patients with mental illnesses in Germany. The present study aims to examine the use of and experiences with e-mental health interventions from the point of view of different types of health care providers for patients with mental illnesses.

**Methods:**

Data from a cross-sectional survey of routine care health care providers in Germany in 2021 were analysed. In this survey, data were collected from *n* = 107 general practitioners (GPs), *n* = 114 specialist doctors, *n* = 102 psychotherapists, and *n* = 102 inpatient clinicians. Assessments included professional use of digital media, as well as knowledge, use and experiences regarding e-mental health interventions in care of people with mental illness.

**Results:**

In the total sample of *n* = 425, 65.6% (*n* = 279) were female. The study participants had an average age of 47.7 years (*SD* = 11.0) and their average work experience was 20.0 years (*SD* = 11.1). Overall, the majority (83.8%, *n* = 353) had heard of e-mental health interventions, but few felt well informed. Only 28.5% (*n* = 121) had already used e-mental health interventions for treatment support. The most commonly recommended e-mental health interventions in the sample were deprexis (39.7%, *n* = 48), moodgym (24.8%, *n* = 30), and iFightDepression (22.3%, *n* = 27). The use was predominantly considered to be helpful and satisfactory. Insufficient knowledge about e-mental health interventions and lack of informational materials for patients were reported as relevant barriers to the use of e-mental health interventions.

**Conclusions:**

E-mental health interventions can be a useful support option, but they are rarely used in the treatment of patients with mental illnesses. There is a need to disseminate information specific to the various types of health care providers. Tailored implementation strategies need to be developed in order to capitalize on the potential of effective e-mental health interventions and to improve health care for patients with mental illnesses.

**Supplementary Information:**

The online version contains supplementary material available at 10.1186/s13690-023-01150-y.

**Table:** 

**Text box 1. Contributions to the literature**
- E-mental health interventions represent innovative and effective adjunct treatment options in the care of people with mental health problems. Practitioners are important multipliers in the scaling of e-mental health interventions
- In order to derive appropriate implementation measures, it is necessary to map current experiences, attitudes, as well as barriers in different mental health care settings
- For the first time, these are illuminated from the perspective of health care providers in different treatment settings in the German routine care of mentally ill people

## Background

More than one-quarter of the adult population in Europe is affected by a mental disorder each year, particularly common are anxiety and depressive disorders [1, 2]. There are unmet needs in the provision and utilization of mental health services around the world [1, 3, 4]. In the EU, less than one-third of people with mental disorders receive adequate treatment [1, 5]. In order to improve mental health care, new and innovative approaches are necessary. In the last two decades, an increasing interest in e-(electronic-) health is reflected in the increasing publication rate on this topic [6].

The term e-mental health refers to the use of information and communication technologies, including digital technologies and new media, for prevention, treatment and aftercare in order to improve mental health and to support mental health services [7]. The potential of e-mental health is increasingly commended and was particularly evident during the COVID-19 pandemic with social distancing rules and limitations on the accessibility of health services [8–10]. An important component of e-mental health is the actual internet and mobile-based interventions that support people with mental disorders, hereinafter referred as “e-mental health interventions”. Other components include video consultation or the integration of virtual reality. An important advantage of e-mental health interventions is their flexibility; they can be used anytime, anywhere and are usually anonymous [11]. The aim of the interventions are changes in behavioral, emotional and cognitive processes and the integration of these skills in everyday life with the help of psychotherapeutic techniques [11, 12]. Most e-mental health interventions employ cognitive behavioral theory (CBT) techniques because the structured nature of the approach lends itself well to self-management. There are a number of scientifically evaluated e-mental health interventions for a variety of mental illnesses, in particular for anxiety disorders, depression, insomnia, posttraumatic stress disorders and obsessive–compulsive disorders [13]. Interventions can be differentiated into guided programs with professional feedback and unguided programs. The effectiveness of both forms of e-mental health interventions has been proven [14–16]. Especially for the improvement of depressive symptoms, there is comprehensive evidence of efficacy [16]. Hence, offering an e-mental health intervention for mild depressive disorders is recommended in the German treatment guideline for depressive disorders [17].

A recent development in Germany is the prescription of digital health applications (DiGA) that will be reimbursed by health insurance, as so-called “app on prescription” [18]. This was implemented by the “Act to Improve Healthcare Provision through Digitalization and Innovation” (Digital Healthcare Act – DVG) in 2019 [19]. DiGA require evidence of effectiveness and are approved as medical devices [18]. To date (February 2023), there are 42 DiGA listed, of which 19 focus on mental health [20]. DiGA are intended to be conducted autonomously by users and to complement conventional treatment options.

Besides DiGA, there are additional effective e-mental health interventions, in particular for depressive disorders, that are operated with the support from health insurances and university institutions [15, 21–23]. The German Association for Psychiatry, Psychotherapy and Psychosomatics (DGPPN) has developed quality criteria to provide practitioners with guidance in choosing a suitable e-mental health intervention [24]. Among other criteria, evidence of efficacy, safety aspects, the qualifications of the developers, and a sufficient description of the intervention are important points of reference [24].

Effective e-mental health interventions are a useful addition to existing treatment services. So far, little is known about whether and how health care providers for patients with mental illnesses use these interventions in practice and what barriers exist. Also, there is little evidence about the extent to which practitioners assess their patients as digitally literate, which is an important prerequisite for recommending an e-mental health intervention [25]. Further, the use of digital technologies in treatment practice can provide an initial indication of the openness to technology of practitioners. Better knowledge of current use, experiences, and attitudes of health care providers for patients with mental illnesses would help to tailor implementation strategies to improve the use of e-mental health interventions in patient care. Therefore, this study aims to fill gaps in research by examining the current use of e-mental health interventions and experiences of different types of health care providers for patients with mental illnesses. In order to provide initial insights into determinants of the use of e-mental health interventions in mental health care, we also aimed to conduct an explorative analysis of the association of practitioner characteristics with the use of e-mental health interventions.

For this purpose, survey data from health care providers from four different health care settings were analyzed. General practitioners (GPs) are often the first and only health care providers to have contact with patients with mental health symptoms [26]. GPs can offer e-mental health interventions as supplemental self-help or to bridge waiting times for an appointment with a psychiatrist or psychotherapist. Another relevant professional group in mental health care are specialist doctors (psychiatrists, psychosomatic specialists, neurological specialists) in outpatient care who could use e-mental health interventions as a complement to psychopharmacological treatment. Third, psychotherapists play a central role in the outpatient treatment of mental disorders in Germany. They can actively integrate an e-mental health intervention into their treatment or offer it to patients on the waiting list [11]. In addition to outpatient care, inpatient settings are another pillar of the German mental health care system. In clinics for the treatment of mentally ill patients, e-mental health interventions can be integrated as a treatment component or recommended for follow-up care [11]. Therefore, inpatient clinicians are included as the fourth professional group surveyed.

The use and experiences in the above-mentioned specialist groups are examined in an explorative approach based on the following research questions:

1. How do health care providers evaluate the e-health literacy of their patients and how are digital media included in patient care?
2. What do health care providers know about e-mental health interventions and how do they currently use e-mental health interventions in treatment of patients with mental illnesses?
3. What are the current experiences and what are potential barriers regarding the use of e-mental health interventions?
4. Which characteristics of health care providers are associated with the recommendation of e-mental health interventions in patient care?

## Methods

This study used cross-sectional data from a survey of health care providers for patients with mental illnesses in Germany. In this study, health care providers from various health care settings for patients with mental illnesses were recruited: GPs, specialist doctors (psychiatric, psychosomatic, and neurological specialists) from outpatient care, psychotherapists from outpatient care, and clinicians from psychiatric and psychosomatic inpatient care. Recruitment took place from March until September 2021. Study documents were sent via post to *N* = 2273 practitioners from the four groups. Openly accessible lists from the Association of Statutory Health Insurance Physicians were used to contact GPs, specialist doctors and psychotherapists. Clinicians were recruited by sending several study documents to clinics. In order to increase the response rate, the health care providers received monetary incentives of 35€ for returning the questionnaire. The response rate was 18.7%; resulting in a sample of *n* = 425 mental health care practitioners (*n* = 107 GPs, *n* = 114 specialist doctors, *n* = 102 psychotherapists, *n* = 102 clinicians). Among the clinicians from inpatient care were *n* = 29 psychologists, *n* = 12 psychotherapists, *n* = 25 physicians in training, *n* = 25 specialists in psychiatry and psychotherapy, *n* = 1 neurologist, and *n* = 19 "others." The latter mostly indicated nursing professions in the free text, as well as occupational therapy and social work.

### Assessments

The health care providers answered a questionnaire that included sociodemographic information, e-health literacy of patients, use of digital media in patient care, as well as knowledge, use, and experiences regarding e-mental health interventions. The questionnaire was based on a previous study by the authors. More detailed descriptions of the development process are published elsewhere [27]. Thus, the questionnaire was based on components of Normalization Process Theory (NPT) and Unified Theory of Acceptance and Use of Technology (UTAUT) [28, 29], which include aspects of coherence, cognitive participation, collective action, and reflexive monitoring [29]. The original questionnaire was adapted for the present study according to results of qualitative interviews with *N* = 21 health care providers for patients with mental illnesses conducted in preparation for the quantitative survey. Accordingly, more specific items for the content of the present study were prepared. A pre-test with *n* = 8 scientific and health care professionals was conducted to evaluate the feasibility and the comprehensibility of the items. The questions relevant for the present study are listed in the appendix.

Socio-demographic information included information on gender (Female/ Male), age in years as well as work experience in years. All other information were assessed using items with categorical response options. As shown in the appendix, multiple answers were possible for some items. The practitioners evaluated the digital health literacy of their patients and were asked about the knowledge of e-mental health interventions to support the treatment of mental illness. We assessed the actual use of e-mental health interventions. We further asked participants who had recommended an e-mental health intervention to specify the e-mental health intervention(s) they recommended, how they integrated the intervention in their treatment, and how many patients they recommended an e-mental health intervention to. In addition, they were asked to rate the effort, patient’s interest, the helpfulness and their satisfaction with the use of e-mental health interventions in mental health care. Practitioners who had not recommended an e-mental health intervention were asked whether they intended to integrate e-mental health interventions in the future and what reasons they had for not recommending an e-mental health intervention.

### Statistical analyses

The statistical analyses were performed using SPSS version 27.0 [30]. Descriptive analyses were conducted for the entire sample and by group and included means (*M*), standard deviations (*SD*) and absolute or relative frequencies with percentages. We further conducted a binary logistic regression analysis with “ever recommended an e-mental health intervention in patient care” as outcome variable and age, gender, professional group, patient-communication via internet, and video-consultation as predictor variables.

## Results

### Socio-demographic information

The sample characteristics are shown in Table 1. The majority of participants were female (65.6%, *n* = 279), the mean age was 47.7 years (*SD* = 11.0). The average work experience was 20.2 years (*SD* = 11.1).

**Table 1:** Sample characteristics

Variable	Total *n* = 425	GPs *n* = 107	Specialist doctors *n* = 114	Psychotherapists *n* = 102	Clinicians *n* = 102
Gender					
Female, *n* (%)	279 (65.6)	63 (58.9)	72 (63.2)	70 (68.6)	74 (72.5)
Age, years, *M* (*SD*)	47.7 (11.0)	50.2 (10.8)	52.7 (8.2)	48.7 (10.4)	38.6 (9.3)
Work experience, years, *M* (*SD*)	20.0 (11.1)	22.5 (11.2)	24.3 (9.0)	20.3 (10.5)	12.0 (11.1)

*Notes. GPs* general practitioners; specialist doctors include psychiatric, psychosomatic, and neurological specialists from outpatient care, clinicians were recruited from psychiatric and psychosomatic inpatient care

### E-Health literacy and digital communication in patient care

**Fig. 1:**
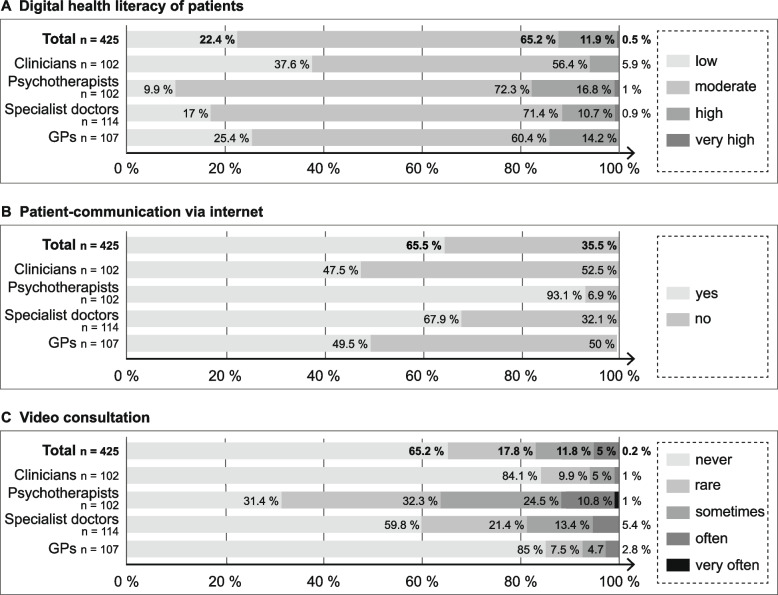
Patient’s e-health literacy and use of digital media in patient care

Figure 1 shows responses regarding digital communication with patients. Most practitioners evaluated the digital health literacy of their patient as moderate (65.2%, *n* = 274). Low digital health literacy of patients was reported by 25.5% (*n* = 27) of GPs and 37.6% (*n* = 38) of the clinicians. In contrast, over 90% (*n* = 91) of psychotherapists rated the digital health literacy of their patients as moderate to very high.

Further, 64.5% (*n* = 272) of the total sample used the internet for communication with patients. This was most common for psychotherapists (93.1%, *n* = 95). Psychotherapists also reported the highest use of video consultations with 36.3% (*n* = 37) using video consultation sometimes to very often. Less than one-fifth of GPs (15%, *n* = 16) and clinicians (14.9%, *n* = 17) used video consultation in patient care.

### Knowledge and use of e-mental health interventions

As Table 2 shows, the majority (83.8%) of practitioners stated having heard about e-mental health interventions. The knowledge was highest among specialist doctors (93.8%) and lowest among GPs (63.6%). With regard to the level of information on e-mental health interventions, most of the practitioners (88.3%) felt uninformed or moderately informed.

**Table 2:** Knowledge about e-mental health interventions

Variable *n* (%)	Total *n* = 425	GPs *n* = 107	Specialist doctors *n* = 114	Psychotherapists *n* = 102	Clinicians *n* = 102
Ever heard of e-mental health interventions					
Yes	353 (83.8)	68 (63.6)	105 (93.8)	92 (91.1)	88 (87.1)
Feeling informed about e-mental health interventions					
Very well informed	1 (0.2)	0	0	1 (1.0)	0
Well informed	48 (11.4)	6 (5.6)	22 (19.6)	9 (8.9)	11 (10.9)
Moderately informed	179 (42.5)	39 (36.4)	52 (46.4)	42 (41.6)	46 (45.5)
Uninformed	193 (45.8)	62 (57.9)	38 (33.9)	49 (48.5)	44 (43.6)
Ever recommended an e-mental health intervention					
Yes	121 (28.5)	10 (9.3)	57 (50.0)	24 (23.5)	30 (29.4)

*Notes. GPs* general practitioners; specialist doctors include psychiatric, psychosomatic, and neurological specialists from outpatient care, clinicians were recruited from psychiatric and psychosomatic inpatient care

Further, 28.5% had recommended an e-mental health intervention to their patients. The use of e-mental health intervention was highest among specialist doctors (50.0%), followed by clinicians (29.4%) and psychotherapists (23.5%). It was lowest among GPs (9.3%).

**Fig. 2:**
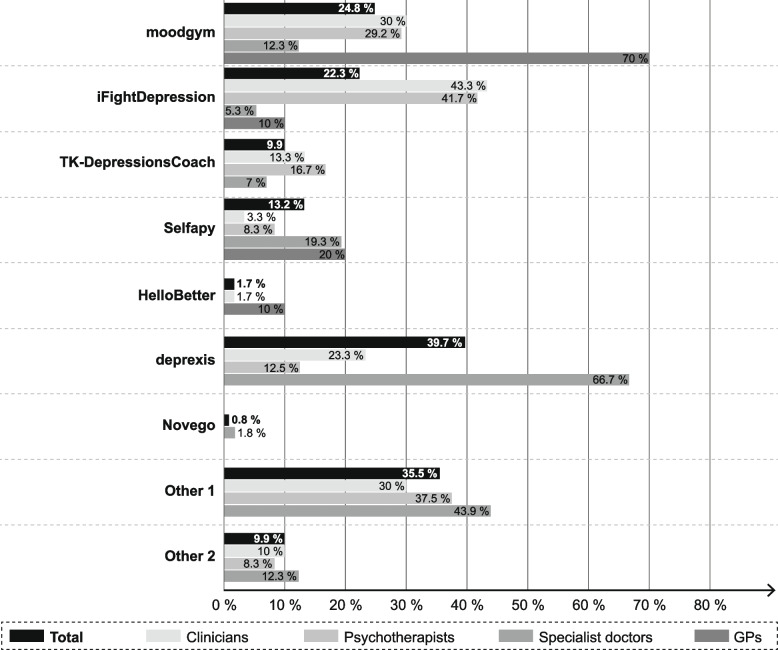
E-mental health interventions recommended by the study sample

Figure 2 shows that the most frequently recommended e-mental health interventions over all groups were deprexis (39.7%), moodgym (24.8%) and iFightDepression (22.3%). With regard to the different types of health care providers, GPs most often recommended moodgym (70%), specialist doctors most commonly recommended deprexis (66.7%), and psychotherapists (41.7%) and clinicians (43.3%) most commonly recommended iFightDepression to their patients. Among others, somnio (*n* = 23), velibra (*n* = 6), invirto (*n* = 3), Mindable (*n* = 1), and NichtraucherHelden-App (*n* = 1) were specified under “Other”.

Table 3 shows the reasons for implementing e-mental health interventions in patient care by practitioners who had experience with such interventions. Most health care providers reported that they had used the e-mental health intervention as supplementary self-help (66.9%), integration into treatment (43.8%) and homework (40.5%). GPs most commonly used it to bridge waiting periods (70%). Most specialist doctors (70.2%), psychotherapists (70.8%) and clinicians (60.0%) recommended e-mental health interventions for supplementary self-help. Half of clinicians recommended use as aftercare (53.3%).

**Table 3:** Use of e-mental health interventions in patient care

Variable *n* (%)	Total *n* = 121	GPs *n* = 10	Specialist doctors *n* = 57	Psychotherapists *n* = 24	Clinicians *n* = 30
Type of use					
Bridge waiting period	45 (37.2)	7 (70.0)	22 (38.6)	7 (29.2)	9 (30.0)
Supplementary self-help	81 (66.9)	6 (60.0)	40 (70.2)	17 (70.8)	18 (60.0)
Integrated into treatment	53 (43.8)	4 (40.0)	23 (40.4)	15 (62.5)	11 (36.7)
Aftercare	27 (22.3)	0	5 (8.8)	6 (25.0)	16 (53.3)
Homework	49 (40.5)	4 (40.0)	21 (36.8)	12 (50.0)	12 (40.0)
Others	4 (3.3)	0	2 (3.5)	1 (0.8)	1 (3.3)
Number of patients					
1 to 5	65 (53.7)	6 (60.0)	30 (52.6)	15 (62.5)	14 (46.7)
6 to 10	29 (24.0)	1 (10.0)	15 (26.3)	4 (16.7)	9 (30.0)
11 to 15	11 (9.1)	1 (10.0)	6 (10.5)	3 (12.5)	1 (3.3)
16 to 20	4 (3.3)	1 (10.0)	1 (1.8)	1 (4.2)	1 (3.3)
More than 20	12 (9.9)	1 (10.0)	5 (8.8)	1 (4.2)	5 (16.7)

*Notes. GPs* general practitioners; specialist doctors include psychiatric, psychosomatic, and neurological specialists from outpatient care, clinicians were recruited from psychiatric and psychosomatic inpatient care. Since the number of GPs with use of e-mental health interventions was small, we omitted reporting relative frequencies

The majority of health care providers (53.7%) recommended an e-mental health intervention to 1 to 5 patients; a quarter (24.0%) to 6 to 10 patients. Finally, 16.7% of clinicians recommended an e-mental health intervention to more than 20 people.

### Experiences and barriers regarding the use of e-mental health interventions

Health care providers who used e-mental health interventions evaluated the effort for the recommendation most commonly as rather low (63.6%, Table 4). This was different for the *n* = 10 GPs, of whom *n* = 5 reported that the effort for recommendation was rather high. Most health care providers reported a moderate interest from their patients in e-mental health interventions (58.7%), and 75.2% said the use of e-mental health interventions was useful to very useful. The majority (64.7%) of health care providers who used e-mental health interventions were rather or very satisfied with the use. While satisfaction was particularly high among clinicians (80%, rather or very satisfied), 42.9% of specialist doctors were less satisfied.

**Table 4:** Experience with e-mental health interventions in routine mental health care

Variable *n* (%)	Total *n* = 121	GPs *n* = 10	Specialist doctors *n* = 57	Psychotherapists *n* = 24	Clinicians *n* = 30
Recommendation effort					
Very high	2 (1.7)	0	1 (1.8)	0	1 (3.30)
Rather high	34 (28.1)	5	18 (31.6)	5 (20.8)	6 (20.0)
Rather low	77 (63.6)	4	34 (59.6)	18 (75.0)	21 (70.0)
Low	8 (6.6)	1	4 (7.0)	1 (4.2)	2 (6.7)
Patient’s interest					
Very high	0	0	0	0	0
High	29 (24.0)	4	12 (21.1)	5 (20.8)	8 (26.7)
Moderate	71 (58.7)	5	32 (56.1)	15 (62.5)	19 (63.3)
Low	21 (17.4)	1	13 (22.8)	4 (16.7)	3 (10.0)
None at all	0	0	0	0	0
The use was…					
Very helpful	4 (3.3)	1	2 (3.5)	0	1 (3.3)
Helpful	87 (71.9)	6	37 (64.9)	18 (75.0)	26 (86.7)
Less helpful	26 (21.5)	3	16 (28.1)	4 (16.7)	3 (10.0)
Not helpful at all	4 (3.3)	0	2 (3.5)	2 (8.3)	0
With the use I’m…					
Very satisfied	6 (5.0)	2	2 (3.6)	0	2 (6.7)
Rather satisfied	71 (59.7)	5	28 (50.0)	16 (69.6)	22 (73.3)
Less satisfied	37 (31.1)	3	24 (42.9)	5 (21.7)	5 (16.7)
Not satisfied at all	5 (4.2)	0	2 (3.6)	2 (8.7)	1 (3.3)

*Notes. GPs* general practitioners. Since the number of GPs with use of e-mental health interventions was small, we omitted reporting relative frequencies

Most health care providers (71.5%) stated that they had not yet recommended an e-mental health intervention in routine care. Of those, the majority (66.4%) stated that they were unsure about the use of e-mental health intervention in future (Table 5). 14.1% stated that they did not want to use them at all. This was most pronounced in specialist doctors (22.8%) and psychotherapists (20.5%). Nearly one in five (19.4%) practitioners reported that they intended to use e-mental health interventions in the future. This was highest in clinicians (25.0%).

**Table 5:** Intention to use e-mental health interventions in the future

Variable *n* (%)	Total *n* = 304	GPs *n* = 97	Specialist doctors *n* = 57	Psychotherapists *n* = 78	Clinicians *n* = 72
Intention of future use					
I would like to use them soon	59 (19.4)	18 (18.6)	8 (14.0)	15 (19.2)	18 (25.0)
I do not want to use them at all	43 (14.1)	12 (12.4)	13 (22.8)	16 (20.5)	2 (2.8)
I don’t know yet	202 (66.4)	67 (69.1)	36 (63.2)	47 (60.3)	52 (72.2)

*Notes. GPs* general practitioners

**Fig. 3:**
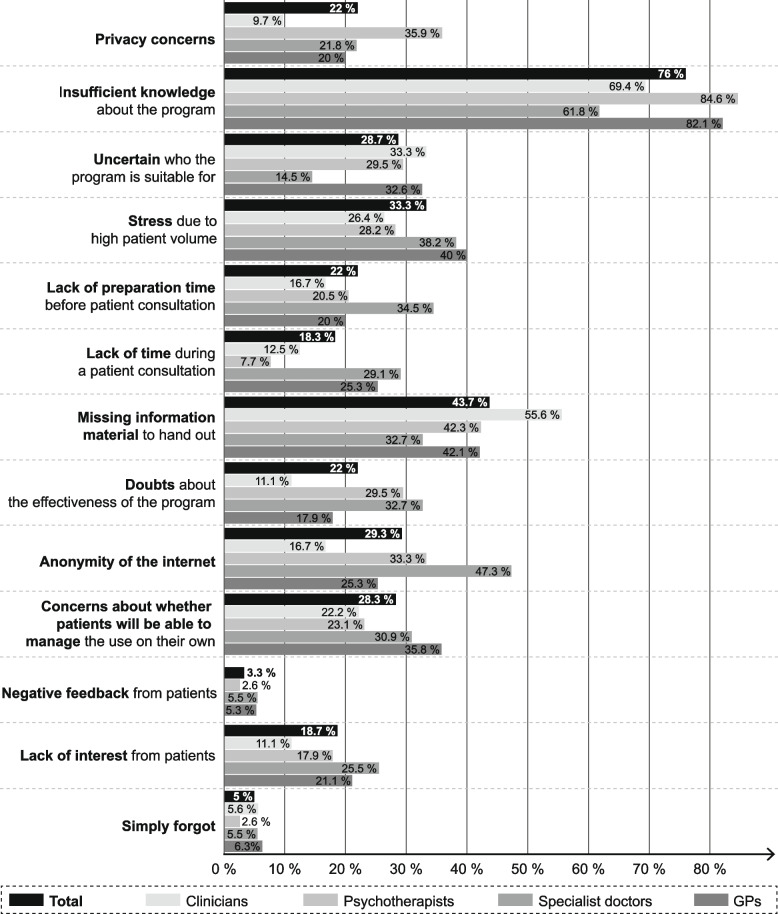
Barriers to using an e-mental health intervention in routine mental health care

Barriers to the use of e-mental health-interventions in those with no experience are shown in Fig. 3. The most commonly reported barriers were insufficient knowledge (76%) and a lack of informational materials to give to patients (43.7%), followed by stress due to high patient traffic (33.3%). Other barriers for health care providers by provider type were: the impersonality of the program for specialist doctors (47.3%), privacy concerns for psychotherapists (35.9%) and uncertainty about for which patients an e-mental health intervention is suitable for clinicians (33.3%).

Table 6 shows the results of the regression analysis. In the binary logistic regression, gender, professional group, patient-communication via internet, and video-consultation were significant predictors of ever having recommended an e-mental health intervention in patient care.

**Table 6:** Binary logistic regression of ever having recommended an e-mental health intervention

Predictor variable	Odds Ratio	95% CI lower bound	95% CI upper bound	*p*
Age	1.009	0.982	1.036	0.523
Gender (ref: Female)				
Male	0.541	0.319	0.918	0.023
Professional group (ref: GPs)				
Specialist doctors	7.486	3.430	16.340	< 0.001
Psychotherapists	1.337	0.563	3.175	0.511
Clinicians	4.604	1.959	10.822	< 0.001
Patient-communication via internet (ref: No)				
Yes	2.545	1.427	4.540	0.002
Video consultation (ref: Never)				
Rare	2.367	1.253	4.473	0.008
Sometimes	2.375	1.121	5.031	0.024
Often	1.796	0.626	5.155	0.276

*Notes*. Binary logistic regression with ‘ever recommended an e-mental health intervention’ as outcome variable and age, gender, professional group, patient-communication via internet, and video-consultation as predictor variables. Since only few participants used video-consultation very often, we subsumed participants who used video-consultation often and very often into one group

*CI* confidence interval, *GPs* general practitioners; specialist doctors include psychiatric, psychosomatic, and neurological specialists from outpatient care, clinicians were recruited from psychiatric and psychosomatic inpatient care

Male health care providers were less likely of having recommended an e-mental health intervention to their patients than female health care providers. The use of e-mental health interventions was significantly higher among specialist doctors and clinicians compared to GPs. Patient-communication via internet predicted higher odds for having recommended an e-mental health intervention to patients. Also, those who used video-consultation rarely or sometimes were more likely to have also used e-mental health interventions compared to those who never used video-consultation.

## Discussion

To the best of our knowledge, the present study is the first to capture the views about e-mental health care from the perspective of different types of health care providers for patients with mental illnesses in Germany. We considered various forms of e-mental health interventions, including apps by prescription (DiGA), as well as free programs and health insurance specific e-mental health interventions, which is a special feature of the present study.

In terms of digital communication in general, psychotherapists reported the highest use, and assigned their patients the highest digital health literacy compared to the other health professionals. This can probably be attributed to regulations for a facilitated use of video consultation in psychotherapy during the COVID-19 pandemic in Germany. In contrast, GPs and clinicians reported that video consultation is not yet an established treatment component. Similarly, the latest data on digitalization of medical and psychotherapeutic practices in Germany show that digital communication and video consultation are common especially among psychotherapists [31]. In international comparisons, other countries are more advanced in e-health and digital patient-provider communication than Germany [32, 33]. For instance, telemedicine and video consultation are firmly anchored in routine care in countries such as Estonia and the Netherlands [32]. Accordingly, the potential of digital communication technologies is not yet fully realized in Germany. Especially with regard to structurally weak regions, in which people with mental illnesses are particularly underserved [34], e-mental health interventions could be a support option.

Due to new laws in Germany such as the “Digital Healthcare Act” and the COVID-19 pandemic, e-health and e-mental health interventions are expected to be on the rise [35]. In our study, 28.5% of health care providers had had experience with e-mental health interventions in treatment of a small number of patients at the time of the survey. Our findings add to the report on digital health applications (DiGA) of a German health insurance, which implies that about 4% of health professionals had prescribed DiGA, with an average number of 2.6 per health professional [36].

Our results also show that the number of practitioners recommending a program and the type of e-mental health intervention varied between different types of health care providers for patients with mental illnesses. Specifically, 50% of the surveyed specialist doctors used e-mental health interventions, but only 10% of GPs. Our results differ from a report issued by the National Association of Statutory Health Insurance Funds [37] which analyzed, among other things, DiGA prescriptions by type of physician. This study found that most DiGA (including those for somatic indications) were prescribed by GPs (32%). Among GPs, the most commonly prescribed DiGA for mental health indications were Selfapy and somnio. Psychiatrists (6%) and psychological psychotherapists (7%) accounted for a smaller proportion of prescribed DiGA and most commonly recommended somnio and deprexis [37].

In our study, the most frequently recommended e-mental health interventions were deprexis (specialist doctors), moodgym (GPs) and iFightDepression (clinicians and psychotherapists). Of those, only deprexis is covered by health insurance. The resulting differences between the studies might be explained by the fact that our results referred exclusively to interventions in the field of mental health and also included freeware programs, in addition to DiGA. This highlights the relevance of non-commercial, free e-mental health interventions such as moodgym or iFightDepression in routine care. Also, to our knowledge, our study is the first to include e-mental health interventions applied in an inpatient context, which adds to previous findings from other studies. In comparing our results to those from the United Kingdom, Breedvelt and colleagues reported much higher usage rates of e-mental health interventions for depression with 72% of GPs recommending them [38]. In our study, only few GPs reported the use of e-mental health interventions for patients with mental illnesses. Wangler (2021) stated that GPs are reluctant in recommending health apps, because they do not feel capable of giving competent advice on the interventions [39]. In line with Breedvelt et al. (2019), GPs can effectively promote the implementation of e-mental health interventions, but more information is needed regarding mental health support.

There are effective e-mental health interventions in Germany and user acceptance is considered high [40]. This is also evident in our study; health care providers evaluated their patient’s e-health literacy and interest in e-mental health interventions to be moderate to high. More than a quarter of health care providers in our sample had already used such interventions in patient care and were satisfied with the experience. This illustrates that e-mental health interventions are relevant to routine care for patients with mental illnesses in Germany and that they can usefully complement conventional treatment options.

Our results further show that most health care providers are already aware of e-mental health interventions, nevertheless they do not feel well informed. With this, our results also demonstrate the unexploited potential of e-mental health interventions in routine care. This is particularly evident when looking at the barriers among health care providers who have not previously used e-mental health interventions in patient care. Of those, two-thirds were unsure whether they could imagine using an e-mental health intervention in future. The most relevant barrier, reported by more than three-quarters of respondents, was insufficient knowledge about e-mental health interventions. Other frequently mentioned barriers were the lack of informational materials to hand out and stress from high patient traffic. Psychotherapists also reported privacy concerns and specialist doctors indicated the impersonality of the programs. The difficulty of responding to individual particularities could be addressed in the development of e-mental health interventions by offering different program versions depending on socio-demographic characteristics or with the inclusion of artificial intelligence.

The results identify the need for information about e-mental health interventions that is tailored to different types of health care professionals. As treatment providers are particularly important multipliers to reaching patients with mental health illnesses, they should receive tailored information. This is in line with findings from Löbner et al. (2022) who concluded from their survey of GPs that awareness about the potential of e-mental health interventions should be raised [27]. Also Hafner et al. (2022) pleaded for a stronger transfer of knowledge into practice [25]. According to our results, implementation strategies should predominantly consider an effective information transfer from research into practice by addressing specific concerns and by developing and disseminating informational materials, such as handouts for patients. Professional organizations can also contribute to this by offering training and information materials. In addition, the specific needs of different types of health care providers must be considered. Our results show that bridging waiting periods is an important issue especially in the primary care setting. Primary care physicians could recommend an e-mental health intervention to their patients with mental health symptoms, helping to provide them with an initial support during the often lengthy search for outpatient psychotherapy. Against this background, the networking of different specialist groups in the care of people with mental illness could be helpful in order to coordinate treatment components from the very beginning. For example, psychotherapists could consult with primary care physicians to determine which intervention is particularly appropriate and can refer to their specific content in treatment. Complementary self-help was relevant to all health care providers but was especially important for specialist doctors. Psychotherapists focused more on the integration of e-mental health interventions into treatment than others did. Finally, follow-up care was particularly relevant in the clinical context. If these specific issues are addressed, implementation of effective online interventions could be improved and patient care could be enriched.

In our study, we examined for the first time which characteristics of health care providers of patients with mental disorders are associated with the use of e-mental health interventions in German routine care. In line with Breedvelt et al. (2019) we found that women were more likely of having used e-mental interventions [38]. This is an interesting finding which deserves further research. As discussed earlier, the results of the regression analysis also illustrate a broader use of digital technologies among specialized doctors and clinicians compared to GPs. It was shown that the use of digital technologies such as digital communication or video consultation was linked to the use of e-mental health interventions. Accordingly, potential positive experiences with digital technologies for communication purposes could increase willingness to recommend e-mental health interventions.

The present study is not without limitations. The response rate to the survey was 18.7%. Although low response rates are to be expected in studies in routine care settings [41], it implies limited generalizability of the study results. Because few GPs had experience with e-mental health interventions, the results in this group should be interpreted with caution. Moreover, the informative value of the results of the exploratory regression analysis is limited to correlative associations. Nevertheless, the results can provide initial indications for the use of e-mental health interventions in practice. Further studies, particularly longitudinal investigations, are pending. Also, the survey took place during the COVID-19 pandemic. The next few years after the pandemic will show whether the advance in digitization will continue to gain momentum after the pandemic. Due to the recent approval of DiGA in Germany, new data is also needed in the short term. In addition, the reasons why the individual profession groups prefer specific e-mental health interventions should be examined in more depth in future studies. Also, other perspectives should be included. While we have focused on different views of health care providers for patients with mental illnesses, future studies could consider the patient perspective.

## Conclusions

Our results show that e-mental health interventions have entered routine care of patients with mental illnesses. Although effective e-mental health interventions exist and legal frameworks aim to facilitate their use, there is more to be done to realize the full potential of effective e-mental health interventions. Further implementation studies are needed to determine how health care providers can better integrate such programs into patient care. Providing effective e-mental health interventions can be a useful complement to existing treatment options and can help to improve care for people with mental illnesses.

## Supplementary Information

**Additional file 1:** **Table A.** Items and response options of the questionnaire.

## Data Availability

The datasets generated and/or analysed during the current study are not publicly available due to privacy but are available from the corresponding author on reasonable request.
