# Supplementary material for: E-mental health in Germany — what is the current use and what are experiences of different types of health care providers for patients with mental illnesses?
Source: Arch Public Health. 2023 Jul 17;81:133. doi: 10.1186/s13690-023-01150-y (PMC10353209; doi:10.1186/s13690-023-01150-y)
Supplement: Supplementary file 1 — Additional file 1: Table A. Items and response options of the questionnaire. [file 13690_2023_1150_MOESM1_ESM.pdf]

Table A. Items and response options of the questionnaire.

**E-Health Literacy and digital media in patient care**
How high do you rate the digital health literacy of your patients?<br><br>Low/ Moderate/ High/ Very high
Are you using the Internet to communicate with your patients?<br><br>Yes/ No
How often do you use video consultations with your patients?<br><br>Never/ Rarely/ Sometimes/ Often/ Very often
**Knowledge and use of e-mental health interventions**
Did you ever hear about e-mental health interventions for treatment support?<br><br>Yes/ No
I feel … about e-mental health interventions for treatment support.<br><br>Very well informed/ Well informed/ Moderately informed/ Uninformed
Have you ever recommended an e-mental health intervention to your patients or integrated it in your patients' treatment?<br><br>Yes/ No
**Questions for those who indicated use of an e-mental health intervention in the past**
Which of the following e-mental health interventions have you ever recommended?[1]<br><br>moodgym/ iFightDepression/ Selfapy/ HelloBetter/ deprexis/ Novego/ Other
How exactly did you integrate the e-mental health intervention in the treatment?[1]<br><br>To bridge the waiting period/ For supplementary self-help/ Integrated into treatment/ As aftercare/ As homework/ Others.
To how many patients did you recommend an e-mental health interventions so far?<br><br>1–5 patients/ 6–10 patients/ 11–15 patients/ 16–20 patients/ More than 20 patients
How do you evaluate the effort of recommending e-mental health interventions as part of your treatment?<br><br>High/ Rather high/ Rather low/ Low
How interested are your patients in applying an e-mental health intervention?<br><br>Very high/ High/ Moderate/ Low/ Not at all
I felt that the use of e-mental health intervention as part of patient care was …<br><br>Very helpful/ Helpful/ Less helpful/ Not helpful at all.
I am … with the use of e-mental health intervention as part of the patient care.<br><br>Very satisfied/ Satisfied/ Rather satisfied/ Not satisfied at all
**Questions for those who did not indicate use of an e-mental health intervention in the past**
To what extent do you intend to use online programs in the future as part of patient care?<br><br>I would like to use them soon/ I do not want to use them at all/ I don't know yet

If you have not used e-mental health interventions, what were the reasons?[1]<br><br>Simply forgot/ Lack of interest from patients/ Negative feedback from patients/ Concerns about whether patients will be able to manage the use on their own/ Anonymity of the Internet/ Doubts about the effectiveness of the program/ Missing informational material to hand out/ Lack of time during a patient consultation/ Lack of preparation time before patient consultation/ Stress due to high patient volume/ Uncertain who the program is suitable for/ Insufficient knowledge about the program/ Privacy concerns/ Other.

Notes. Selfapy, HelloBetter, deprexis, and Novego were DiGA at the time of the survey, moodgym and iFightDepression were free e-mental health interventions.

[1] Multiple response options were possible.
